# Do *Grapholita funebrana* Infestation Rely on Specific Plum Fruit Features?

**DOI:** 10.3390/insects10120444

**Published:** 2019-12-11

**Authors:** Roberto Rizzo, Vittorio Farina, Filippo Saiano, Alberto Lombardo, Ernesto Ragusa, Gabriella Lo Verde

**Affiliations:** 1 CREA-Research Centre for Plant Protection and Certification, SS.113, Km 245,5, 90011 Bagheria, PA, Italy; roberto.rizzo@crea.gov.it; 2 Department of Agricultural, Food and Forest Sciences, University of Palermo Viale delle Scienze, 90128 Palermo, Italy; vittorio.farina@unipa.it (V.F.); filippo.saiano@unipa.it (F.S.); ernesto.ragusa@unipa.it (E.R.); 3 Engineering Department, University of Palermo, Viale delle Scienze, 90128 Palermo, Italy; alberto.lombardo@unipa.it

**Keywords:** plum fruit moth, Tortricidae, cultivar susceptibility

## Abstract

The effective control of the plum fruit moth, *Grapholita funebrana* (Lepidoptera: Tortricidae) still represents a difficult challenge for organic plum farming. Little information is available on the susceptibility of plum cultivars to this moth pest. We investigated the roles of several fruit parameters (i.e., shape, volume, hardness, fruit colour, and physiochemical properties) on the susceptibility of four different plum cultivars (Angeleno, Friar, President and Stanley) to *G. funebrana* attack. Field data demonstrated the importance of some fruit parameters (i.e., elongation index, sugar degree, titratable acidity, cover colour percentage) on susceptibility to *G. funebrana* infestation. Under laboratory conditions, colour and shape had a significant role in determining the time spent on false fruits, i.e., female moths preferred yellow and rounded fruits over elongated red or green fruits. Angeleno (yellow and rounded fruits) and Stanley (green and elongated fruits) were the most and least susceptible cultivars, respectively. Overall, this study adds useful knowledge about plum cultivar susceptibility to *G. funebrana.* Information reported here may be useful to improve integrated pest management strategies both in conventional and organic orchards because the use of less susceptible cultivars may reduce insecticidal treatments, limiting the development of resistance in target insects and the harmful side effects on beneficial species.

## 1. Introduction

Plums (*Prunus domestica* L. and *Prunus salicina* Lindl.) are widely cultivated in areas with a temperate and subtropical climate in China, India, America, Japan, and European countries [1,2,3]. In 2017, world plum production reached almost 12 million tons [4]. Plums are commonly used as fresh as well as processed fruits (jams, compotes, dehydrated plums, and juices). Plum fruits appear to provide a variety of health benefits, due to their high-quality constituents, like carbohydrates, vitamin A, vitamin C, calcium, magnesium, iron, potassium, and antioxidants [5,6,7,8].

Due to the economic and social importance of plum production, several studies evaluated new varieties adapted to regional climatic conditions, displaying resistance or tolerance to abiotic and biotic stresses (among them diseases and pests), improved fruit quantity, and overall quality [1,3,9,10,11,12,13]. Nevertheless, a detailed examination on the discrimination of cultivars is lacking, as prior studies have been often carried out at regional level or regarded specific quality parameters [14,15,16,17,18]. An evaluation of the susceptibility of different plum cultivars to the main pests can provide useful information for plum improvement programs and pest management strategies.

*Grapholita funebrana* Treitschke (Lepidoptera Tortricidae), the plum fruit moth (PFM), is one of the main pests of plum orchards in Europe. This species is oligophagous and attacks different host plants within the family Rosaceae, such as the fruits of plums, cherries, and peaches. This moth can complete one or two generations per year in many parts of central and eastern Europe [19] and three generations per year in Italy [20,21]. As with other moths, females of PFM attract mates using pheromone signals [22,23,24] and lay eggs on developing fruits of host plants [25]. Neonate larvae typically bore into immature fruits, where they feed and develop until pupation. Damage is due to the feeding activity of the larvae inside the fruits, which causes changes in fruit colouration, early ripening and fruit fall. Furthermore, infested fruits show penetration holes made by neonate larvae, which are characterised by the presence of gum, and exit holes made by mature larvae leaving the fruit [26]. Both result in reduced marketability and significant yield decreases. In conventional agriculture, this pest is primarily controlled by the use of broad-spectrum insecticides [19]. In organic plum orchards, good fruit protection can be achieved by alternating a low number of spinosad applications and mineral oil applications, despite the short period of effectiveness typical of spinosad [27].

In other tortricid species, the effect of the cultivar in the oviposition choice has been assessed in field studies for *Lobesia botrana* (Denis and Schiffermüller), *Eupoecilia ambiguella* (Hübner) *Grapholita molesta* (Busck), and *Cydia pomonella* (L.) [28,29,30,31]. In laboratory tests, preference for different apple cultivar was demonstrated for *C. pomonella* [32], whereas the influence of fruit characteristics like shape, size, texture and colour on *L. botrana* and *E. ambiguella* have been studied [33]. Moreover, differences in *L. botrana* performance (larval development time, fecundity, egg size and egg hatchability) due to grape cultivar have been investigated [34,35,36].

The oviposition behaviour of lepidopterans and the selection of an egg-laying site depends upon the multimodal integration of sensory cues in the host location and selection processes. This complex process involves visual cues, among them physical characteristics (e.g., the shape, size, and colour) of leaves, fruits, stems, and other plant parts, and chemical cues [37,38,39,40,41,42,43]. In particular, the effects of physical, chemical, and visual cues on the host plant choice have been studied with the aim of increasing the efficiency of pheromone traps for catching pests, and the results have demonstrated that lepidopteran catches can be improved by manipulating the visual aspect of the trap design [44,45,46,47,48].

Although some plum cultivars have been observed to vary in their susceptibility to PFM [21,27,49], the factors affecting the infestation level have not yet been investigated. In this study, our objective was to assess the influence of some fruit parameters, in particular, the shape and colour of surface of plum fruits, on the susceptibility of four different plum cultivars (Angeleno, Friar, President, and Stanley).

## 2. Materials and Methods

### 2.1. Study Area, Sampling Plane, and Infestation Level

The research was performed in two Sicilian plum orchards located at San Giuseppe Jato, Italy (37°59′36″ N, 13°13′29″ E and 37°59′42″ N, 13°12′20″ E, respectively). The two orchards had regular shapes and surfaces of 6 and 5 ha, respectively. The trees were managed using routine organic cultural practices. In both orchards, 8-years-old plum trees grafted on Mirabolano rootstocks and trained to a vase shape were planted in single rows, spaced at 6 m between rows and 3 m between trees inside the row. In the two orchards, four groups of eight rows, containing an average number of 90 trees, are present. Each cultivar is distributed along two rows in each group of rows. Four cultivars were chosen for the study: Friar and Angeleno (*Prunus salicina* Lindl.) and Stanley and President (*Prunus domestica* L.). For each cultivar, three blocks of ten trees, randomly placed in the orchards, were used: two of them were located in the first orchard and the third in the second one. This setting was needed in order to include a sufficient number of trees, in which a homogenous number of fruits of similar sizes were present.

Three plum trees were marked in each of the 3 blocks per cultivar and used for fruit sampling that aimed to record both the PFM field infestations (2012–14) and physical parameters (2013–14, Data Set 1). On these trees, at each sampling date, 8–10 fruits per tree (72–90 per cultivar) were collected randomly around the canopy. Fruit sampling was carried out every 10–14 days starting from the detection of the first eggs on the fruits up to the last week of July: from 4 May to 24 July 2012, from 5 May to 29 July 2013, and from 5 May to 30 July 2014. In 2012, the cultivar Friar was not sampled due to inadequate fruit production. All fruits were then kept in the Department of Agricultural, Food, and Forest Science (University of Palermo, Palermo, Italy). After measuring biometrical and physical parameters, fruits were dissected under a stereomicroscope to record the presence of PFM larvae. A fruit was considered infested when larvae or their penetration and exit holes were present.

In all years, four pheromone-baited (dodecyl acetate (50%), Z8-dodecenyl acetate (49%), and E8-dodecenyl acetate (1%), Isagro Italy) sticky traps were placed in each orchard in the last ten days of March (26 March 2012, 22 March 2013, and 24 March 2014) and were checked every 7–10 days. Field observations to detect eggs on fruits started after the first males were caught in the traps (9 April in 2012 and 2013, 4 April in 2014).

### 2.2. Physical and Chemical Properties of Fruits

Fruits from Data Set 1 were assessed for biometrical and physical parameters as follows: weight, using a digital scale (KERN 440 d = 0.1 g, Kern & Sohn GmbH, Balingen, Germany); longitudinal and transversal diameters, using an SPI Digimax Digital Caliper (model 30-440-2; Swiss Precision Instruments, Garden Grove, CA, USA); and hardness by analogical penetrometer (RG Strumenti, Parma, Italy) with an automatic depth limiter and a tip of 2 mm in diameter. The longitudinal and transversal diameters of fruits were then used to calculate the elongation index of each fruit (as the ratio between them), and the fruit volume was calculated with the following formula: (4/3) *π* longitudinal diameter * transversal diameter^2^.

In both years, on each sampling date, an additional forty fruits were collected from four unmarked trees of the same blocks to be analysed for colour and chemical properties (Data Set 2). In order to avoid any influence of infestation on chemical parameters, only uninfested fruits were sampled, i.e., fruits in which the absence of eggs, penetration, or exit holes was assessed in the field using a magnifying glass. To evaluate colour, fruits were kept in the laboratory. A fruit analysis system (FAS) based on digital images was used to determine the percentage and intensity of colour [50]. Each group of fruits sampled on the same tree was photographed with a digital camera, obtaining digital images that were used to determine the percentage and intensity of peel red colour. Specifically, we used an algorithm that converts images from RGB to CIE (Commission Internationale de l’Eclairage) in the L*a*b* format, which extracts the fruit from the image (removing the image background) and quantifies colour characteristics as the weighed distance of each pixel in the image from a reference sample (best coloured area interactively chosen from a well coloured fruit). For plum fruits, characterised by a red-purple cover colour, a green-red threshold algorithm was used to obtain a separation of the total fruit area (number of pixels) into two sub-regions, cover colour (closer to red-purple) and ground colour (closer to green). The pixel ratio was used to quantify cover colour as a percentage of the total fruit area. The output is an index for the cover colour (CCI) ranging from 0 (not red-purple) to 1 (red-purple), a ground colour index (GCI) ranging to 0 (green) to 1 (not green). The pixel ratio between GCI and CCI was used to quantify the cover colour percentage (CCP) [50,51].

On the same fruits, after the photos were taken, the following chemical parameters were recorded: total soluble solid (TSS, as °Brix) by a digital refractometer (Atago Palette PR-32, Atago Co., Ltd., Tokyo, Japan); titratable acidity (TA, as g/L of malic acid); and pH using a CrisonS compact titrator (Crison Instruments, SA, Barcelona, Spain). For Data Set 2, an average value of the variables was obtained per each tree at each sampling date.

### 2.3. Laboratory Trials

PFM adults were reared from infested fruits of the Sanacore cultivar for use in laboratory trials. They were collected in July 2014 from an organic plum orchard at San Giuseppe Jato (37°59′50″ N, 13°12′45″ E, Palermo Province, Italy). The cultivar Sanacore, different from those used in the following tests, was chosen to avoid any possible pre-imaginal conditioning.

The pupae were sexed and kept separately in plastic cages (30 × 20 × 30 cm) under a L16:D8 photoperiod at a constant temperature (26 ± 1 °C) and relative humidity (65%–70% RH) [52].

Because the flight performance of PFMs is greater in fed adults [53], moths were supplied with a 10% honey-water solution (g/mL). Mated females were obtained by placing two males and two females (<24 h old) in the same cage for 2 days before each test. The moths were used only once and were not exposed to plant-related odour sources before the trials. In order to assess the effect of fruit shape and colour, three sets of trials were performed using false fruits made of clay (air dried modelled clay Dawi^®^ Classic) and entirely coloured (Acrilic Master Ferrario^®^, Ferrario S.P.A. Bologna, Italy). The trials were conducted under the same conditions described above (cage sizes 30 × 20 × 30 cm, L16:D8 photoperiod, 26 ± 1 °C temperature and 65%–70% RH). Based on the characteristics of the cultivars studied in the field trials, two fruit shapes and three different colours were tested. The fruit shapes were rounded (minimum diameter 3.2 cm and maximum diameter 3.8 cm) and elongated (minimum diameter 3.2 cm and maximum diameter 4.1 cm), whereas the tested colours were red (red deep 013—R = 185.34, G = 49.26, B = 50.05), yellow (primary yellow 004—R = 245.54, G = 217.48, B = 0), and green (chromium oxide green 036—R = 84.96, G = 116.47, B = 78.87). In the first trial, nine rounded false fruits (three for each colour) were used, whereas, in the second trial, nine elongated false fruits (three for each colour) were used. In the third trial, 18 false fruits were used, three for each of the six combinations of shape/colour (yellow/rounded, yellow/elongated, red/rounded, red/elongated, green/rounded, and green/elongated). False fruits were placed on the floor of the cage, and the position of the different shaped/coloured false fruits was randomly changed for each replicate. Fruits were placed on the floor of the cage at about the same distance each other and from cage walls. Six replicates were performed for each trial. In each replicate, one mated female (4 days old) was introduced into the cage containing the false fruits, observed for 120 min, and then removed. The periods of time (minutes) spent on each false fruit (positive responses) or on the cage walls (negative responses) were recorded.

### 2.4. Statistical Analysis

As infestation recorded in May was almost null, all the analyses were performed using data from June and July. The first analysis of the field data (Data Set 1) was conducted to determine any difference between the four cultivars with respect to fruit infestation. Data from the three years (2012–2014) were used in a binary logistic regression analysis, without obtaining a reliable estimation of the model. Therefore, a general linear model (GLM) was applied. In order to meet the assumptions of normality, the square root transformation was applied to the percentage of infested fruits recorded from each tree. The model included cultivar, date (nested in year), block (nested in cultivar and year), and tree (nested in block) as factors. Each year was analysed separately as the inclusion of all three years in the model was not estimable. Residual analysis, performed for each year, resulted as not significant, confirming that the root square transformed data leads to meet the normal assumption (Anderson–Darling test: 2012, AD = 0.497, *p* = 0.209; 2013, AD = 0.453, *p* = 0.268; 2014, AD = 0.563, *p* = 0.143). The infestation analysis found block and tree to be not significant (2012: Block F_9,143_ = 1.12, *p* = 0.384; Tree F_24,143_ = 0.92, *p* = 0.581; 2013: Block F_12,143_ = 1.13, *p* = 0.367; Tree F_44,143_ = 1.06 *p* = 0.412; 2014: Block F_8,167_ = 1.85, *p* = 0.163; Tree F_12,167_ = 1.03, *p* = 0.428), and hence, these variables were removed from subsequent models. Removal of these variables allowed us to use the binary logistic model to evaluate the impact of cultivar and date on the probability of infestation. Comparisons between infestations of cultivar pairs were obtained according to the Bonferroni correction, with a familywise error rate equal to 0.95.

Fruit variables (volume, elongation, hardness and weight—Data Set 1) were singularly analysed using GLM. The model for each variable included: percentage of infestation (as a metric variable), cultivar, year, tree (nested in block), block (nested in cultivar and year), and date (nested in year), and the interaction between cultivar and year. As the block of trees remains the same in each year but changes from one year to another, the block and, consequently, the tree have been included as random factors. In order to meet the assumption of GLM (normality of errors), a suitable transformation was applied to each variable. Volume, elongation, and weight were log-transformed as indicated by the Box-Cox procedure, while no transformation was required for hardness. After assessing that infestation and block were not significant for all parameters (infestation: volume F_1,2833_ = 1.35, *p* = 0.245; weight F_1,2833_ = 1.49, *p* = 0.222; elongation index F_1,2832_ = 0.002, *p* = 0.963; hardness F_1,2833_ = 0.002, *p* = 0.965; block: volume F_1,2833_ = 0.80, *p* = 0.706; weight F_1,2833_ = 0.68, *p* = 0.827; elongation index F_1,2832_ = 0.75, *p* = 0.759; hardness F_1,2833_ = 1.07, *p* = 0.415), the analyses were repeated excluding infestation and block as factors, and including both infested and uninfested fruits.

Fruit colour and chemical properties (GCI, CCI, CCP, TSS, TA, and pH—Data Set 2) were analysed using GLM with cultivar, year, and date (nested in year) as variables. As CCP and CCI changed rapidly over time, a reliable model was not attainable. As such, they were removed from this analysis and used only for the following ones.

Finally, to evaluate the capability of the recorded variables for cultivar identity and to analyse the influence of all variables on the infestation, an Overall Data Set was used. It was obtained by adding to Data Set 2 the variables means from four trees of Data Set 1. The four trees were randomly chosen among those included in Data Set 1 from the same blocks of the corresponding trees sampled in Data Set 2. Merging data from the two data sets was possible on the basis of the previous analyses, which showed no significant effect of block and tree on infestation and no significant effect of infestation on the fruit parameters from Data Set 1.

A discriminant analysis was performed, aimed at evaluating the predictive multivariate capability of the recorded fruit parameters for cultivar identity. Data were standardised, as usual, subtracting mean and dividing by standard deviation, in order to eliminate the different units among variables, and the correlation matrix was used.

Infestation analysis was performed using the binary logistic regression and applying a stepwise procedure in order to eliminate redundancy due to the large multicollinearity of variables, if any.

As the discriminant analysis showed a high predictive capability of the fruit variables on cultivar determination, in order to assess the effect on the infestation of those variables along with cultivar and date, three binary logistic models were performed. The first model included only fruit variables. The second one included only date and cultivar. A third one, the most comprehensive model, included all factors (date, cultivar, fruit parameters), and was used as a benchmark for the other two.

The laboratory results were analysed using only positive responses (i.e., number of minutes spent on false fruits). For trials 1 and 2, in which only rounded or elongated false fruits were used, respectively, the effect of colour was assessed by analysing the percentage of time spent on each false fruit with a 1-way ANOVA, followed by Tukey’s HSD test. The analysis of residuals ascertained that the normality hypothesis was acceptable in both trials 1 and 2 (trial 1, elongated fruits: Anderson Darling = 0.467, *p* = 0.205; trial 2, rounded fruits: Anderson Darling = 0.479, *p* = 0.200). Data from the third trial, in which the false fruits were different in both shape and colour, were analysed using a GLM in which the two factors (shape and colour) and the interaction between them were considered. Also in this case, the analysis was confirmed by a residual analysis of normality (Anderson Darling = 0.654; *p* = 0.08).

All statistical analyses were done using MINITAB software (Minitab, Inc., State College, PA, Italy).

## 3. Results

### 3.1. Field Infestation and Physical-Chemical Properties of the Fruits

**Figure 1:**
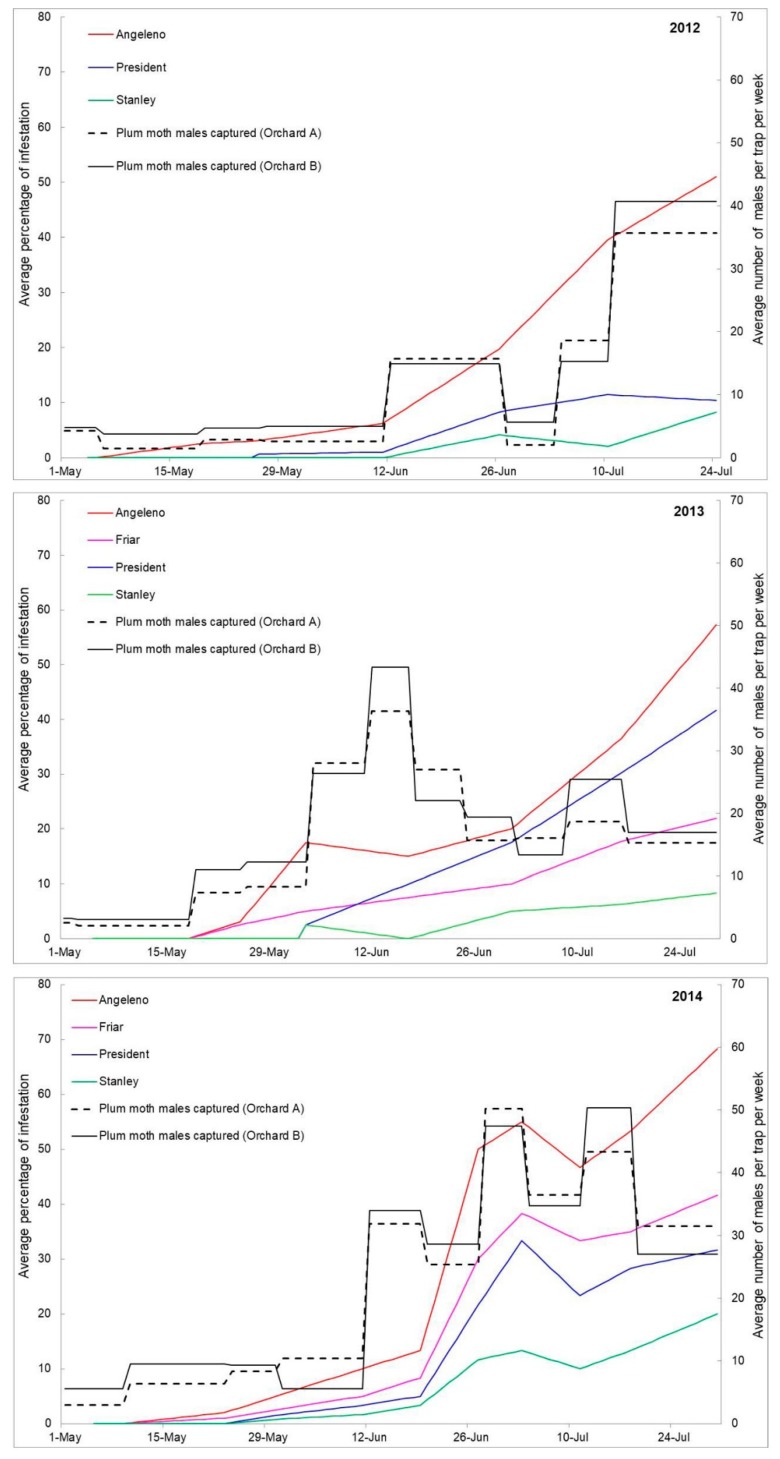
Infestation trend recorded on the four plum cultivars in the three years 2012–2014, and the mean number of Plum Fruit Moth males caught in pheromone traps placed in the two plum orchards.

In Figure 1, the infestation trend of the three years, together with the mean number of PFM males caught in pheromone traps placed in the two orchards, are reported. In all years, the number of PFM males in the traps placed in the two orchards was quite similar. Infested fruits were recorded starting from 28 May 2012, 20 May 2013, and 23 May 2014, respectively (Figure 1). In all years and during all the sampling period, Angeleno and Stanley were the most and the least infested cultivars, respectively.

These observations were confirmed by the results of the binary logistic regression (Table 1). Significant differences in the estimated probability of infestation among the four cultivars were found within each year (Bonferroni correction with familywise error rate equal to 0.95). In all years, both date and cultivar factors were significant (Table 1). Angeleno and Stanley were the cultivars that always significantly differed, while Friar and President showed intermediate infestation levels, always significantly differing from Angeleno and Stanley.

**Figure 2:**
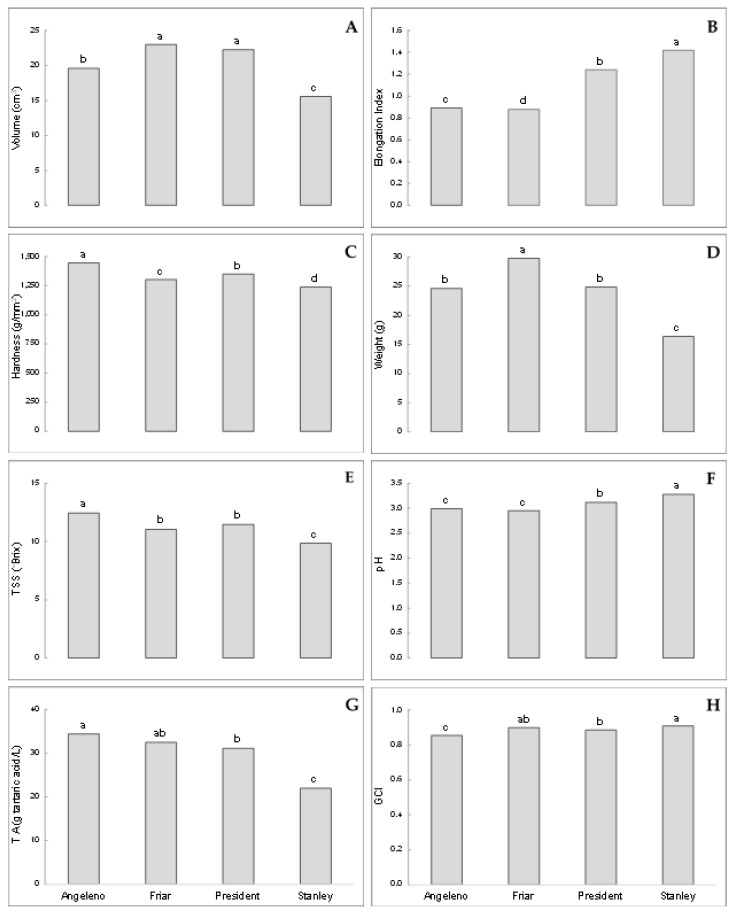
Average values of the variables for which significant differences among the cultivars occurred (different letters indicate significant differences among the four cultivars, general linear model (GLM) followed by Tukey’s HSD test, *p* < 0.001). Volume (**A**), elongation index (**B**), hardness (**C**), and weight (**D**) were analysed using the Data Set 1, whereas Data Set 2 was used for total soluble solid (TSS) (**E**), pH (**F**), titratable acidity (TA) (**G**), and ground colour index (GCI) (**H**).

With regards to the fruit characteristics, an overall evaluation of the four variables (Data Set 1) highlighted the occurrence of significant differences among the cultivars (volume F_(3;2927)_ = 65.88, *p* < 0.001; elongation index F_(3;2927)_ = 4619.53, *p* < 0.001; hardness F_(3;2927)_ = 71.14, *p* < 0.001; weight F_(3;2927)_ = 149.95, *p* < 0.001; TSS F_(3;191)_ = 74.54, *p* < 0.001; pH F_(3;191)_ = 32.33, *p* < 0.001; TA F_(3;191)_ = 70.32, *p* < 0.001; GCI F_(3;191)_ = 33.75, *p* < 0.001). Fruit shape showed significant differences among the four cultivars (Figure 2B). Angeleno and Friar are characterised by an elongation index lower than 1 (rounded fruits), while President and Stanley by an elongation index higher than 1 (elongated fruits). The Stanley fruits resulted in the smallest and the most elongated (Figure 2A,B). Differences among the four cultivars were also found in the analyses performed with the Data Set 2 for the variables TSS, pH, TA, and GCI (Figure 2E–H). Stanley differed from all the other cultivars for the lowest values of TSS and TA (Figure 2E,G), and the highest values of pH (Figure 2F). In general, the only two cultivars that resulted significantly different for all considered parameters were Angeleno and Stanley, whereas Friar and President showed intermediate values for most of the considered variables (Figure 2A–H).

Results from GLM performed on the variables included in Data Set 1 showed that the factors year, cultivar, date, cultivar * year, and tree were significant (Table 2). On the contrary, the preliminary analysis performed for infestation showed that tree was not significant. This could be due to the fact that fruits are infested by insects moving preferentially on neighbouring trees inside the same block, while biometric characteristics are typical of each tree.

Looking at the importance of each factor within the same parameter measured by means of F-values (Table 2), date was the predominant factor for volume, weight, and hardness, as expected, because these variables are strongly related to fruit growing. On the contrary, cultivar resulted in being the predominant factor for elongation index, as expected, considering that fruit shape is typical of each cultivar.

The result of the discriminant analysis, conducted using the standardised variables (Overall Data Set), revealed that, even with a cross-validation, the proportion of correct attribution is very high (Table 3). Misclassifications occurred for Angeleno (two fruits placed in the Friar group), Friar (six fruits placed in the Angeleno group), and President (one fruit placed in the Stanley group). Elongation index was the most relevant parameter in the cultivar attribution, whereas the other ones had a lower influence. These results lead to the conclusion that the recorded variables reliably described the cultivars under study. Therefore, these variables represent a good starting point for the following analyses about infestation.

With regard to the binary logistic models, performed to evaluate the dependency of the infestation on the predictive factors considered in the study (Overall Data Set), the three resulted models showed a good predictive capability. The model including only fruit variables (not reported), reached the lowest predictive capability (R^2^-adj = 63.69%, AIC = 1731.98) and resulted so complex that any biological interpretation was not possible, as it included a lot of factors and interactions. On the contrary, the overall model (Table 4) reached the highest predictive power (R^2^-adj = 72.14%, AIC = 1688.70) and included as a single factor, among fruit parameters, only TA, whereas the elongation index, TSS, and CCP, besides cultivar and date, were included within the second-order factors (interactions terms).

It should be observed that fruit parameters included in the model can be related to PFM females behaviour during fruit choice for oviposition (fruit shape and CCP) or to fruit suitability for larval development (TSS and TA). The model that included only date and cultivar, in spite of its simplicity, reached an intermediate predictive capability (R^2^-adj = 66.81%, AIC = 1711.50). Both date and cultivar significantly influence infestation (Table 5), whereas the interaction between the two factors was not significant. Comparing the last two models, in which all factors (date, cultivar, fruit parameters) and only date and cultivar were included, respectively, it is evident that the lack of information of the simpler model did not dramatically reduce its predictive capability. Therefore, the predictability of infestation without knowing cultivar and date, but on the base of fruit parameters, could be obtained only at the cost of an unmanageable model.

All of the goodness-of-fit tests of the reported models were found to be non-significant.

### 3.2. Laboratory Trials

**Figure 3:**
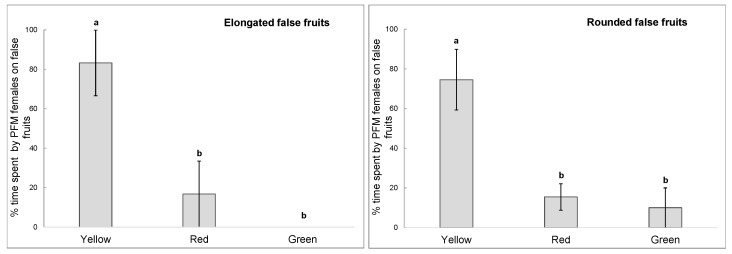
Percentages of time spent by the Plum Fruit Moth females on false fruits of different colours in trials 1 and 2, in which only elongated or rounded fruits were used, respectively. Means with same letter are not significantly different (ANOVA followed by Tukey’s HSD test).

PFM females spent a significantly greater percentage of the time on yellow false fruits in the trials 1 and 2, in which only rounded or elongated false fruits were used, respectively. The insect spent 83% of time on yellow elongated false fruits (trial 1) and 75% on yellow rounded false fruits (trial 2). In both trials, percentage of time spent on red and green false fruits were not significantly different (1-way ANOVA: trial 1, elongated fruits: F_(2,9)_ = 10.50, *p* = 0.004; trial 2, rounded fruits: F_(2,12)_ = 10.21, *p* = 0.003, Figure 3).

**Figure 4:**
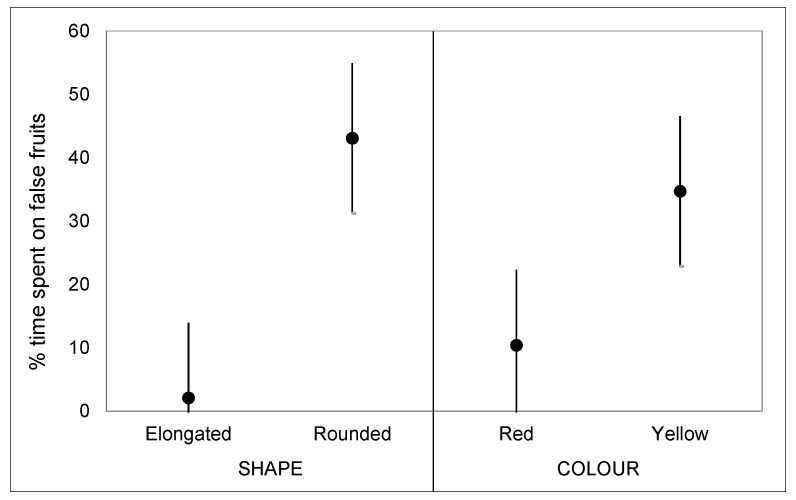
The mean percentages of time spent by the Plum Fruit Moth females on the false fruits in the trial in which the false fruits of different colours and shapes were used.

In the third trial, the false fruits were different both in shape and colour, and—as it was possible to see at first glance—the time percentage spent on elongated or rounded green false fruits are all quite near to zero. This fact made it impossible to formulate an overall model able to reliably describe the effects of the considered factors on the insect choice; therefore, we decided to exclude the results from green false fruits in subsequent analysis. A GLM, applied on response data for red and yellow fruits, showed a not significant effect of the interaction shape/colour (shape/colour F_(1,20)_ = 3.13 *p* = 0.09), while a significant effect was found for shape (F_(1,20)_ = 12.96, *p* = 0.002) and colour (F_(1,20)_ = 4.56, *p* = 0.045). Specifically, rounded and yellow false fruits were preferred by females compared with all the other combinations shape/colour (Figure 4).

## 4. Discussion

In general, the visual recognition of host trees by phytophagous insects involves several factors, among which are the perceptions of chromatic stimuli or other physical signals, such as size and shape. Colour can also influence the response to a suitable substrate for egg laying. Different insect species are sensitive to different colours. This aspect has been studied for flower-visiting insects, like bees or butterflies [54,55], or in carpophagous flies [56,57,58]. The role of shape in selecting oviposition site was investigated in fruit flies, suggesting that shape is, together with size and colour, an attractive factor in *Rhagoletis pomonella* (Walsh), *Rhagoletis cerasi* (L.), *Ceratitis capitata* (Wiedemann), and *Bactrocera oleae* (Rossi) [59,60,61,62,63,64].

With regard to tortricid moths, most of the studies on the attractive effect of shape and colour are aimed at optimising the effectiveness of catch devices [65]. For example, the attractiveness of white to *C. pomonella* is twice that of blue [66]. For *Heliothis armigera* (Hübner), the green colour is more attractive than the brown, whereas *G. molesta* shows oviposition preferences for dark red fruits [28], and *Cydia strobilella* L. catches were higher in red traps compared to black, white, or orange ones [67]. In *Argyrotaenia montezumae* (Walsingham), a higher number of eggs per female was laid on green, blue, and yellow substrates than on transparent and pink ones [68]. The shape of the oviposition site can influence selection in *Choristoneura fumiferana* (Clemens) [69], whereas in laboratory tests, *L. botrana* and *E. ambiguella* females preferentially laid eggs on spherical surfaces [33,70].

Our field results indicated an association between the shape/colour combination and other fruit parameters that are apparently not perceivable by ovideponent females. Most of the fruit parameters recorded in the present study had significant differences among fruits from the four cultivars (Figure 2), and the discriminant analysis showed a high proportion of correct classification for fruits of the different cultivars (Table 3). Differences among the four cultivars were also found for infestation levels recorded in the three study years (Table 1), confirming the results obtained in previous field studies [21,27]. Thus, colour and shape play an essential role in the fruit choice for oviposition and seems to be related to a combination of other parameters not directly detectable by the insect, like TSS and TA (Table 4), which contribute to making fruits suitable for larval development. Moreover, even if fruit parameters were able to effectively define the cultivars, the higher explanatory power obtained in the analysis performed by also including, besides fruit parameters, cultivar and date indicates that some other unidentified factors can be involved in the relationship between fruit from different cultivars and the PFM females, which leads to the oviposition choice. In *L. botrana*, differences in the sugar content and acidity were associated with differences in larval development of different grape cultivars [71], and sugar importance was confirmed in studies on the different composition of artificial diets [72,73]. In the present study, sugar content showed significant differences among the plum cultivars, as also found for Serbian plums [18].

The remarkable differences found between Angeleno and Stanley in susceptibility to PFM infestation were related mainly to differences in shape, colour, sugar content, and acidity. Despite the sampling date significantly influenced all variables associated with fruit growth, as expected (Table 2), the physiological ripening stage could play only a secondary role. In fact, Angeleno, which always shows the highest infestation level, is classified in Southern Italy as “late ripening cultivar”, as ripening occurs in the second half of September, 65 days after the reference cultivar, Shiro, which ripens in the first decade of July [74]. On the other hand, Stanley is classified as “medium ripening cultivar”, as in southern Italy, ripening occurs from 14 to 25 August [74]. In our case, differences in infestation level between the two cultivars were observed from first sampling dates up to harvest (Figure 1), which was carried out before the physiological ripening stage of the fruit.

Laboratory trials confirmed that both fruit shape and colour affect the host-finding behaviour of PFM females. Specifically, there was a clear preference for yellow fruits with a spherical shape. These results are consistent with field results, as Angeleno, which is characterised by rounded yellow fruits, was the most infested cultivar, while Stanley, whose fruits are elongated and green, was the least infested cultivar by the PFM.

In general, the choice of a suitable substrate or host for oviposition in terms of nutritional and chemical qualities plays a key role in the survival and completion of the different life stages of lepidopteran insects [28,75,76,77,78]. Moreover, female preference for “good quality hosts” seems to be stronger in oligophagous insects than in polyphagous ones [79]. This is particularly true for those species, like PFM, in which larvae enter the fruit, feed on its pulp, and remain inside the fruit until the pre-pupal stage because they are usually incapable of moving from one fruit to another.

## 5. Conclusions

The combined evaluation of all parameters investigated in the present study, resulted in a comprehensive explanation of the differences among the cultivars in terms of susceptibility to PFM infestation, whereas laboratory results highlighted the relevant role of shape and colour of plums in the fruit choice by the moth females.

The present study represents a first step in the assessment of the susceptibility of the studied plum cultivars to PFM and of the factors connected to that susceptibility. Further studies could identify other unknown factors (i.e., volatile compounds), which are involved in the relationship between fruit from different cultivars and the PFM females and, consequently, in the oviposition choice. Moreover, the cultivar effect and the role of physiochemical fruit parameters on larval performance could be interesting issues to be investigated in future studies.

Despite that the use of resistant cultivars is strongly recommended in integrated pest management programs [80], genetic improvement programs that aim to identify sources of plum resistance to PFM are lacking, probably due to the high efficiency of the control methods commonly applied, like the use of chemical insecticides [81,82] and mating disruption [83,84].

## Figures and Tables

**Table 1:** Mean infestation percentages in the different plum cultivars in 2012–2014. Different letters indicate significant differences between cultivars within the same year, as resulted from the estimated binary regression model. A significant effect of date and cultivar on infestation is shown by the results of the model (** means *p* < 0.001, NS means not significant).

Cultivar	2012	2013	2014
Angeleno	29.17 a	35.58 a	42.38 a
Friar	-	15.06 b	27.38 b
President	7.81 b	25.96 c	20.95 b
Stanley	3.65 c	5.45 d	10.48 c
	Binary logistic regression results		
Date	χ^2^ = 77.19 ** (DF 3)	χ^2^ = 84.28 ** (DF 4)	χ^2^ = 179.54 ** (DF 7)
Cultivar	χ^2^ = 126.83 ** (DF 2)	χ^2^ = 113.07 ** (DF 3)	χ^2^ = 133.73 ** (DF 3)
Goodness-of-fit tests	χ^2^ = 717.17 NS (DF 1146)	χ^2^ = 1075.70 NS (DF 1240)	χ^2^ = 1598.95 NS (DF 1670)

**Table 2:** The F-values resulting from GLM analyses, singularly performed on each fruit parameter of the Data Set 1, showing the importance of each significant factor (*p* < 0.001) within fruit parameters.

Factors	DF	F-Values			
		Volume	Weight	Hardness	Elongation Index
Cultivar	3	56.97	115.23	78.89	3983.68
Year	1	26.84	19.34	16.27	67.77
Cultivar * Year	3	15.24	10.86	33.16	12.92
Date (Year)	10	351.92	422.61	309.50	72.45
Tree (Cultivar, Year)	76	5.18	5.75	2.11	3.34
Total DF	2927				

**Table 3:** Classification of fruits from different cultivars compared with the groups determined by the discriminant analysis performed on all parameters. Coefficients show the impact of the different parameters in the correct attribution to the four cultivars.

**Put into Group**	**True Group**			
	**Angeleno**	**Friar**	**President**	**Stanley**
Angeleno	46	6	0	0
Friar	2	42	0	0
President	0	0	47	0
Stanley	0	0	1	48
Total No.	48	48	48	48
No. correct	46	42	47	48
Proportion	0.96	0.88	0.98	1.00
	**Linear Discriminant Function for Cultivar**			
	**Angeleno**	**Friar**	**President**	**Stanley**
Constant	−19.15	−25.79	−8.67	−42.48
Volume	−8.29	−15.73	9.23	14.80
Elongation Index	−41.91	−49.12	28.19	62.84
Weight	8.66	16.56	−8.28	−16.94
Hardness	2.36	1.70	−0.90	−3.16
Titratable Acidity	−2.72	−4.73	3.08	4.37
pH	−1.59	−1.53	0.76	2.37
Total Soluble Solid	0.21	1.03	−0.12	−1.12
Ground Color Index	1.39	3.94	−1.73	−3.60
Cover Color Index	−4.25	−5.02	2.07	7.19
Cover Color Percentage	1.26	−0.03	−1.09	−0.15

**Table 4:** The overall model obtained by including all the binary logistic analysis factors (date, cultivar, and fruit parameters, Overall Data Set). The model shows which factors significantly affect the infestation level.

Source	DF	Adj Dev	Adj Mean	Chi-Square	*p*-Value
Regression	21	424.67	20.22	424.67	<0.001
Titratable Acidity	1	15.26	15.26	15.26	<0.001
Elongation Index * Cultivar	3	30.29	10.10	30.29	<0.001
Total Soluble Solid * Cultivar	3	27.29	9.10	27.29	<0.001
Titratable Acidity * Date	11	166.21	15.11	166.21	<0.001
Cover Colour Percentage * Cultivar	3	16.06	5.35	16.06	<0.001
Error	170	134.91	0.79		
Total	191	559.58			

**Table 5:** The model obtained including only date and cultivar in the binary logistic analysis (Overall Data Set). Results show that both factors, but not their interaction, significantly affect the infestation level.

Source	DF	Adj Dev	Adj Mean	Chi-Square	*p*-Value
Regression	14	387.90	27.71	387.87	<0.001
Cultivar	3	176.60	58.85	176.56	<0.001
Date	11	229.30	20.84	229.27	<0.001
Error	177	171.70	0.97		
Total	191	559.60			
